# Glossary

**Published:** 1995

Amnesia: Memory loss.
Amygdala: A *gray-matter* structure in the front portion of the *temporal lobe*.
Anterior: Toward the front of the body.
Anterograde amnesia: Inability to remember new information for more than a few seconds; anterograde amnesia is a primary characteristic of *Korsakoff’s syndrome.*
Atom: Once believed to be indivisible, atoms are the primary unit of matter composing *elements.* An atom consists primarily of a *nucleus*, which contains protons and neutrons, and electrons that orbit the nucleus. The number of protons, neutrons, and electrons is different for each element. An atom of a specific element is the smallest unit that exhibits all the characteristics and properties of that element.
Axial: Relating to or situated in the central part of the body. In imaging, an axial image is one that is obtained by rotating around the axis of the body.
Axon: Part of a neuron consisting of a single fiber that carries nerve impulses from the *neuron* to other cells.
Basal ganglia: A group of *gray-matter* structures at the base of the *cerebral hemispheres* that are involved in motor control.
Caudate nucleus: A curved mass of *gray matter* that is part of the *basal ganglia* and protrudes into the lateral *ventricle*. The caudate nucleus plays a major role in voluntary motor activity.
Cell body: The part of a nerve cell that contains the *nucleus*.
Cerebellum: The structure at the base of the brain that is involved in the control of muscle tone, balance, and sensori-motor coordination.
Cerebral cortex: The outer layer of *gray matter* covering the *cerebrum*. The cortex contains areas for processing sensory information and for controlling motor functions, speech, higher cognitive functions, emotions, behavior, and memory.
Cerebral hemispheres: The two halves of the *cerebrum* that comprise the *cerebral cortex*, the underlying white matter, and the *basal ganglia*. Each hemisphere primarily controls the sensory input and motor functions of the opposite half of the body.
Cerebrospinal fluid (CSF): The clear fluid that fills the cavities (i.e., *ventricles*) within the brain and that surrounds the brain and spinal cord.
Cerebrum: The largest portion of the brain; includes the *cerebral hemispheres*.
Compound: In chemistry, a substance composed of two or more different *elements* that are chemically combined.
Corpus callosum: The tract of nerve fibers connecting the two *cerebral hemispheres.*
Cortex: The outer layer of an organ.
Dementia: A condition of global intellectual impairment, including the loss of abstract thinking and memory, personality changes, breakdown of social skills, and other disturbances of higher cognitive functioning.
Dendrite: The branched projections of a *neuron* that receive nerve impulses from other cells. Most neurons have more than one dendrite.
Diencephalon: The area of the brain located beneath the *cerebral cortex* consisting of the *thalamus* and the *hypothalamus*.
Element: A substance composed of only one kind of *atom*.
Fissure: A deep *sulcus*.
Frontal lobes: The *anterior* region of the *cerebrum.*
Gray matter: Brain tissue composed mostly of *dendrites* and *cell bodies* that makes up the outer surface of the *cerebral cortex* as well as portions of the brain at the base of the *cerebral hemispheres.*
Gyrus (Gyri): The ridges of rounded, convoluted brain tissue that forms the *cerebral hemispheres.*
Hippocampus: A region of the *temporal lobe* that is thought to play a role in learning and memory as well as in alcohol withdrawal seizures.
Histology: The science of the detailed structure of cells, tissues, and organs in relation to their function.
Hypothalamus: An important part of the limbic system with many regulatory functions, including the control of motivation and emotional behavior. The hypothalamus is located in the *diencephalon*.
Inferior: In anatomy, situated nearer to the bottom (i.e., in humans, toward the soles of the feet).
Isotope: An isotope is one of two or more *atoms* that have the same number of protons (i.e., are chemically identical) but have different numbers of neutrons.
Korsakoff’s syndrome: An organic brain syndrome associated with prolonged, heavy ingestion of alcohol, characterized by *anterograde amnesia*. Also see *Wernicke-Korsakoff syndrome (WKS)*.
Lenticular (lentiform) nuclei: Part of the *basal ganglia* comprising the putamen and the globus pallidus. Two sets of lenticular nuclei exist, one in each hemisphere of the brain.
Magnetic field: A physical field that arises from an electric charge in motion or from a magnet, producing a force that attracts particles of specific *elements*.
Mammillary body: A paired brain structure located near the *hypothalamus* that is involved in memory and in the control of autonomic (i.e., involuntary) body functions.
Morphology: The biological study of the form and structure of organisms.
Nucleus: The center structure of a cell or of an *atom*.
Neuron: A nerve cell, which is made up of a *cell body*, an *axon*, and one or more *dendrites*.
Neurotransmitter: A chemical messenger released by a *neuron* to carry a signal to adjacent neurons.
Occipital lobe: The part of the *cerebrum* at the rear of each *hemisphere*, separated from the *parietal lobe* by the parieto-occipital *sulcus;* the surface of the occipital lobe is involved in vision.
Orbitofrontal cortex: The part of the *cerebral cortex* covering the base of the *frontal lobes.*
Parietal lobe (region): The region of the *cerebral cortex*, located in the middle part of the *cerebral hemispheres*, that mainly receives information not from the sensory organs but from receptors in or near the body surface. It is separated from the *occipital lobe* by the parieto-occipital *sulcus*.
Posterior: Toward the rear of the body.
Prefrontal cortex: The most *anterior* section of the *frontal cortex;* involved in memory processes, specifically in delayed response tasks.
Radio wave: An electromagnetic wave having a frequency between approximately 10 kilohertz and 300,000 megahertz; a key component of imaging technology.
Radioisotope: An *isotope* that changes to a more stable state by emitting particles (i.e., radiation) from its *nucleus*.
Receptor: A structure in the wall or interior of a nerve cell or other cell that recognizes and binds to *neuro-transmitters* and other chemical messengers.
Sulci (Sulcus): The grooves or furrows on the surface of the brain.
Superior: In anatomy, situated nearer to the top of the head.
Sylvian fissure: The deepest and most prominent of the *fissures* in the *cerebral cortex.*
Temporal lobe: The region of the *cerebral cortex* forming part of the sides and bottom of the brain on each side. This region is involved in sensory processing, language functions, and emotions.
Thalamus: The *gray-matter* structure that forms part of the *diencephalon*, the brain’s relay center to the *cerebral cortex.*
Thiamine: Vitamin B_1_; a deficiency in this vitamin has been linked to *Korsakoff’s syndrome*.
Tomography: The technology of making an image of thin slices of tissue within the body.
Ventricles: A normal cavity (e.g., in the brain or heart). In the brain, the ventricles are filled with *cerebrospinal fluid.*
Vermis: The structure located between the two halves of the *cerebellum* that is important for controlling particular motor functions.
Wernicke-Korsakoff syndrome (WKS): A neurological disorder thought to be caused by *thiamine* deficiency. WKS is characterized by impairments in memory (e.g., *anterograde amnesia*) as well as deficits in abstraction and problem-solving. Wernicke’s encephalopathy is an acute condition characterized by general confusion and incoherent speech. It may or may not precede *Korsakoff’s syndrome.*
White matter: Brain tissue composed mainly of *axons*.

**Figure:**
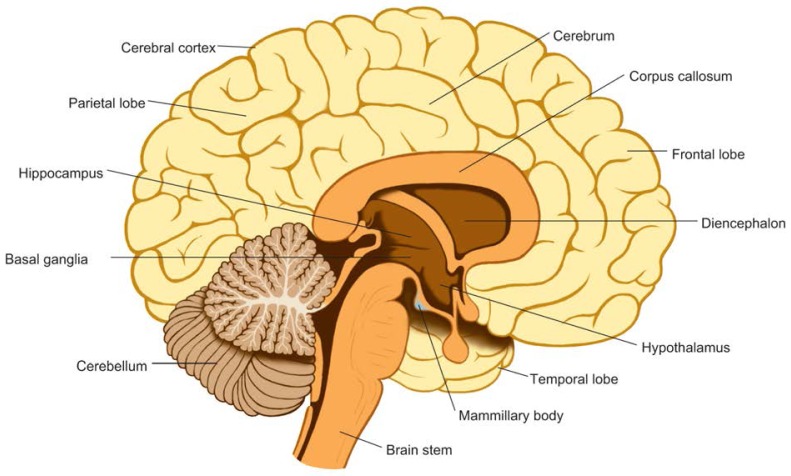
Schematic drawing of the brain.

**Figure:**
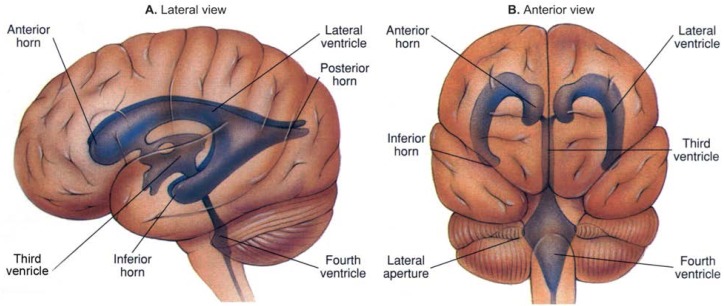
Two views of the human brain showing the location of the ventricles.

**Figure:**
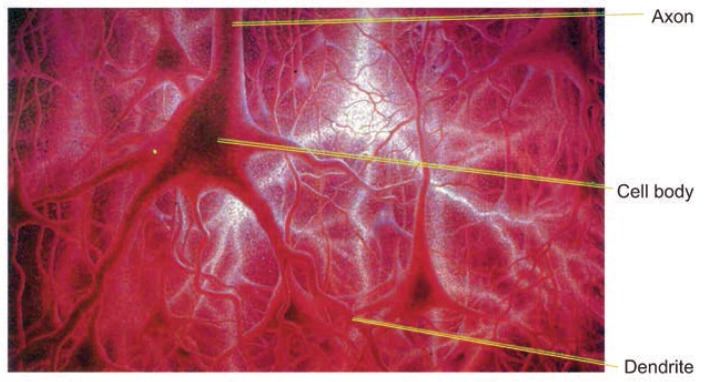
Schematic drawing of the structure of neurons.

